# Dihydroxynaphthalene‐based mimicry of fungal melanogenesis for multifunctional coatings

**DOI:** 10.1111/1751-7915.12347

**Published:** 2016-02-02

**Authors:** Jong‐Rok Jeon, Thao Thanh Le, Yoon‐Seok Chang

**Affiliations:** ^1^ Institute of Agriculture & Life ScienceGyeongsang National UniversityJinju52727Korea; ^2^ School of Environmental Science and EngineeringPOSTECHPohang37673Korea

## Abstract

Material‐independent adhesive action derived from polycatechol structures has been intensively studied due to its high applicability in surface engineering. Here, we for the first time demonstrate that a dihydroxynaphthalene‐based fungal melanin mimetic, which exhibit a catechol‐free structure, can act as a coating agent for material‐independent surface modifications on the nanoscale. This mimetic was made by using laccase to catalyse the oxidative polymerization of specifically 2,7‐dihydroxynaphthalene. Analyses of the product of this reaction, using Fourier transform infrared‐attenuated total reflectance and X‐ray photoelectron spectroscopy, bactericidal action, charge‐dependent sorption behaviour, phenol content, *Zeta* potential measurements and free radical scavenging activity, yielded results consistent with it containing hydroxyphenyl groups. Moreover, nuclear magnetic resonance analyses of the product revealed that C‐O coupling and C‐C coupling were the main mechanisms for its synthesis, thus clearly excluding a catechol structure in the polymerization. This product, termed poly(2,7‐DHN), was successfully deposited onto a wide variety of solid surfaces, including metals, polymeric materials, ceramics, biosurfaces and mineral complexes. The melanin‐like polymerization could be used to co‐immobilize other organic molecules, forming functional surfaces. In addition, the hydroxyphenyl group contained in the coated poly(2,7‐DHN) induced secondary metal chelation/reduction and adhesion with proteins, suggesting the potential of this poly(2,7‐DHN) layer to serve as a platform material for a variety of surface engineering applications. Moreover, the novel physicochemical properties of the poly(2,7‐DHN) illuminate its potential applications as bactericidal, radical‐scavenging and pollutant‐sorbing agents.

## Introduction

For a material to achieve effective adhesion and coating on a solid surface, it requires functional moieties that can strongly bind to the substrate. Ideally, these groups should be capable of forming diverse bonds with several different kinds of surfaces and, once the initial coating has been assembled, the binding should be durable against external stresses such as exposure to water and contamination with various substances. Toward this end, polymers that contain dihydroxyphenyl groups (i.e., catechols) have been shown to effectively coat a variety of surfaces (Lee *et al*., 2006, 2007, 2011; Zhao and Waite, 2006; Waite, 2008). For example, polydopamine, which contains catechols, has been deposited on many types of solids, providing a useful platform for surface functionalization (Lee *et al*., 2007). Reactive quinone groups derived from the catechol groups of polydopamine can undergo secondary reactions such as Michael addition and Schiff base reactions. In combination with the material‐independent coating capabilities of polydopamine, these reactions allow for a variety of applications, including film filler modification (Phua *et al*., 2013), water treatment (Lee *et al*., 2012), lithium ion batteries (Ryou *et al*., 2011), drug delivery (Cui *et al*., 2012), cell culture/differentiation (Shin *et al*., 2012), artificial cell encapsulation (Yang *et al*., 2011), biosensors (Lynge *et al*., 2011) and carbon nanotube modification (Wang *et al*., 2011).

Catechol conjugation has also been shown to have the capability to functionalize natural and synthetic polymers. The adhesive action, biocompatibility and hydrophilicity of such polymeric materials can be controlled by modifying the catechol structure and hence its physicochemical properties (Yamada *et al*., 2000; Lee *et al*., 2011; You *et al*., 2011; Cho *et al*., 2013). The catechol‐containing species can also be modified with polyphenolic moieties in order to affect its binding of diverse materials (McDonald *et al*., 1996; Jeon *et al*., 2009, 2010, 2013a; Sileika *et al*., 2013). This finding strongly supports the idea that the previously reported catechol‐based adhesives, including those whose adhesive action derives from polydopamine coating and catechol conjugation, could be reproduced by materials displaying polyphenolic moieties.

Polyaromatics that contain hydroxyphenyl groups are widespread in nature. These *in vivo* natural matrices are generally synthesized by biotransformation of phenol groups into the corresponding radicals, followed by oxidative polymerization via repeated coupling processes catalysed by laccases. For instance, plants use the anabolic action of laccase on monolignols or flavonoids to synthesize plant components such as lignin and polyflavonoids (Jeon *et al*., 2012). Insects employ similar actions with catechol derivatives for morphogenesis, leading to formation of their exoskeletons (Jeon *et al*., 2012). Fungi also use laccase enzymes in conjunction with 1,8‐dihydroxynaphthalene (DHN) and DOPA for their melanogenesis (Jeon *et al*., 2012; Jeon and Chang, 2013b).

In the present study, 2,7‐DHN and fungal laccase were employed to mimic fungal melanogenesis (Eisenman and Casadevall, 2012) and to test whether the strong binding affinities displayed by polycatechol products could be reproduced by other kinds of polyphenolic structures. 2,7‐DHN does not possess a catechol moiety at all in its structure, and catechol groups are thus not present in the product of the laccase‐catalysed polymerization of 2,7‐DHN. First, we evaluated whether poly(2,7‐DHN) exhibited adhesion to several different solid surfaces and characterized the structural and physicochemical properties of poly(2,7‐DHN). We then carried out immobilization experiments with a coated layer of poly(2,7‐DHN) and demonstrated that the polymeric layer could act as a platform for a variety of surface engineering purposes.

## Results and discussion

**Scheme 1:**
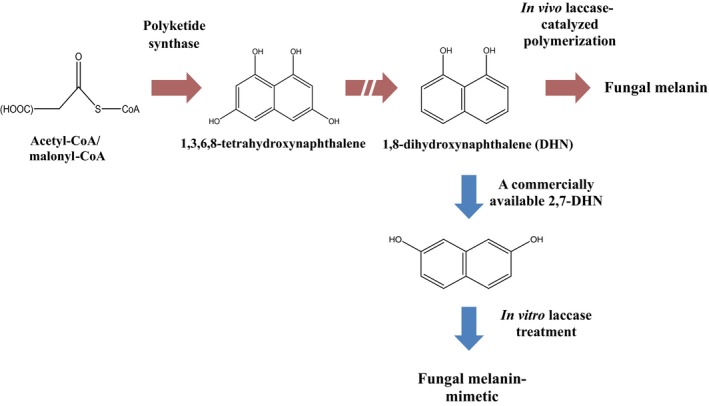
Scheme for *in vivo* dihydroxynaphthalene (DHN)‐mediated fungal melanogenesis pathway. The pathway indicates that laccase oxidative action on DHN leads to fungal melanogenesis. We thus employed *in vitro* laccase‐catalysed oxidation of 2,7‐DHN, the commercially available DHN, to mimic fungal melanogenesis.

**Figure 1:**
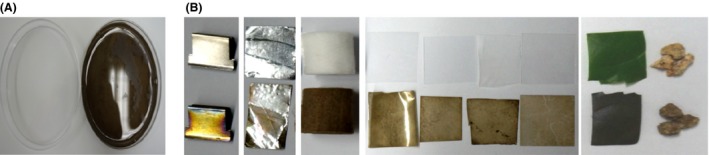
One‐pot modification of solid surfaces through *in vitro* laccase‐catalysed polymerization of 2,7‐DHN. A. Photograph of dipping media before and after polymerization. Left to right: before polymerization; after polymerization. B. Left to right: stainless steel; aluminium; cellulose acetate; casted polypropylene; PET; nylon; glass; plant leaf; granite. Top to down: without dipping; with dipping.

The anabolic action of laccase on 1,8‐DHN *in vivo* is known to lead to fungal melanogenesis, as shown in Scheme 1. To test the feasibility of *in vitro* polymerization of DHN, we reacted purified fungal laccase with commercially available 2,7‐DHN, using acidic sodium acetate buffer to maximize the enzymatic activity. The colouration demonstrated in Fig. 1A clearly indicates that the *in vitro* conditions allowed for efficient catalytic oxidative polymerization of 2,7‐DHN. As previously reported (Jeon *et al*., 2012; Jeon and Chang, 2013b), laccase should transform the phenolic moieties of the DHN into the corresponding quinone, followed by a coupling process. The coupling results in polymerization of the DHN, with its conjugated aromatic rings contributing to the formation of a chromophore that absorbs visible light.

Laccases are generally known to catalyse the formation of single bonds during coupling processes. We thus hypothesized that laccase would also catalyse the formation of single bonds between 2,7‐DHN monomers and set out to determine whether the theoretically predicted *m/z* values of 2,7‐DHN oligomers can be detected experimentally. The results obtained by direct electrospray ionization‐mass spectrometry (ESI‐MS) indicated the presence of 2,7‐DHN oligomers. MS/MS patterns of the candidate *m/z* values were also found to overlap with those of standard 2,7‐DHN, consistent with the oligomers being derived from 2,7‐DHN (Table S1, Fig. S1). Together with the significant colour change, these results demonstrated the feasibility of using the coupling action of laccases in our experimental conditions.

We next employed prep‐liquid chromatography (LC) and nuclear magnetic resonance (NMR) for detailed structural elucidation of the metabolites. Only those products obtained within 2 hours of the start of the reaction were separated by LC because longer reaction times induced a tight assembly of the products, which prevented dissolution in conventional organic solvents. We found two major peaks (at 3.2 and 7.8 min in the given separation condition) from the prep‐LC, and the components in these peaks appeared to be more hydrophobic than 2,7‐DHN (data not shown). NMR analysis of these components clearly demonstrated that our laccase reaction led to C‐C or C‐O coupling between 2,7‐DHN monomers (Fig. S2). C‐O coupling would result from nucleophilic attack of phenoxyl radicals onto either the 3‐ or 6‐carbon position of the naphthalene ring followed by a release of hydrogen ions, whereas C‐C coupling would result from electron delocalization from the phenoxyl radicals to naphthalene carbons before the coupling processes. Notably, the above bond formation mechanisms hardly affect the position of the hydroxyphenyl groups in 2,7‐DHN during such enzymatic polymerization, strongly suggesting that catechol structures found in mussel adhesive proteins (MAPs) are not formed in our products.

It is well known that fungal melanin can bind to several organic and inorganic substances (Purvis *et al*., 2004; Martinez and Casadevall, 2006). This binding ability is known to be linked to the bioremediation potential and anti‐fungal drug resistance of melanized biomatrices in fungi. Fungal melanin can adsorb several metal species such as uranium and copper. Fungal cell wall‐localized melanin can also capture drugs, thus preventing their anti‐fungal action. These phenomena motivated us to determine whether the enzymatically synthesized poly(2,7‐DHN), which exhibits structural similarity with DHN‐based fungal melanin, could be deposited onto different kinds of solid substrates and form nanothick layers. Notably, *in situ* incubation of solid substrates such as metals, polymeric materials, ceramics, biosurfaces (e.g. plant leaf) and mineral complexes (e.g., granite) with poly(2,7‐DHN) resulted in significant colour changes of the solid surfaces (Fig. 1B). Since the colour is derived from the polymerized 2,7‐DHN, the surface dyeing indicated that this fungal melanin mimetic was able to bind to solid surfaces in a material‐independent manner. Such non‐specific binding has been previously reported for mussel‐inspired polycatechol structures including polydopamine and polynorepinephrine (Lee *et al*., 2007; Kang *et al*., 2009; Lynge *et al*., 2011). We also performed scanning electron microscopy (SEM) analyses with polystyrene‐based plastic surfaces; as expected, dramatic change in surface morphology was observed because of the attachment of poly(2,7‐DHN) (Fig. S3).

While previous reports have shown material‐independent adhesion of plant‐related polyphenolics onto different kinds of solid surfaces (Jeon *et al*., 2013a; Sileika *et al*., 2013), with the structures of these polyphenolics containing adhesion‐promoting catechol groups, our results demonstrate that material‐independent adhesion could also be achieved with hydroxyphenyl groups. Our results may have been due to the synergistic effects of the polyaromaticity and multiple hydroxyphenyl groups of poly(2,7‐DHN): the hydrophobicity of the aromatic structures may have contributed to a decrease of solubility in water, hence promoting binding to surfaces, while the functional groups may have exerted adhesive forces similar to those of polycatechols.

The deposition rate of poly(2,7‐DHN) onto a polyethylene terephthalate (PET) film was such that a layer 75 nm thick was deposited after 15 hours of incubation (see Experimental section and Fig. S4 for the method used to measure coating thickness), suggesting that this fungal melanin mimetic is able to perform surface modification on the nanoscale. We further measured the time course of changes in the coating thickness. The kinetics of the coating layer growth as described in Table S2 indicated the initially assembled layers to be stable at the nanoscale, that is, without any significant increase of the thickness into the microscale. In addition, the water contact angles of the film surfaces (i.e., of casted polypropylene, nylon and PET) changed upon coating, indicating that the characteristics of the surfaces were significantly modified by the coating action (Table S3). It is noteworthy that the modification (i.e., of the contact angle of water) resulting from poly(2,7‐DHN) deposition depended on the type of solid surface. This result suggests that the assembly of the polymerized structures on the surfaces would be affected by surface characteristics such as surface energy and roughness. Indeed, differences in contact angle with surface characteristics have been reported for polynorepinephrine (Kang *et al*., 2009), although not to the same degree as for poly(2,7‐DHN).

**Figure 2:**
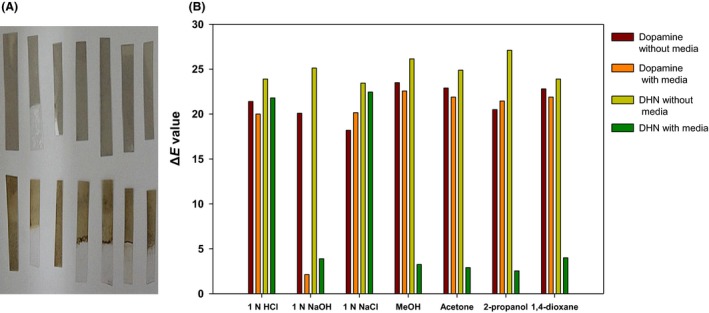
A. Dopamine (top) and 2,7‐DHN (down) coated PET. Half region of each PET film was soaked in the corresponding media for 10 s. B. Colour value change of the coated PET films before and after the soaking. The colour value (ΔE) of PET film was calculated using the equation: ΔE = [(100 − L*)^2^ + (a*)^2^ + (b*)^2^]^1/2^.

The applicability of a coated layer depends on its robustness against external stresses because one of the main purposes of coating is the protection of the surfaces to be coated. The robustness of the coated layers was therefore evaluated by employing a PET film, which is frequently used in the coating industry. The layers were seen to exhibit excellent resistance against 1 N hydrochloric acid and 1 N sodium chloride, but treatment with either 1 N alkaline solution or organic solvents (i.e., methanol, acetone, 2‐propanol and 1,4‐dioxane) led to an immediate disruption of the polymeric matrix, followed by complete detachment of the coating (Fig. 2A and B). These results indicate that the poly(2,7‐DHN) was relatively stable under mild conditions but not under harsh alkaline and organic solvent conditions. The immediate detachment of the enzymatically synthesized poly(2,7‐DHN) from the film strongly supports the proposal that the sorption between the polymeric material and the solid surfaces resulted from non‐covalent interactions rather than from covalent linkages between them.

The observation that the assembly of the polymer, which controls the porosity of the melanin‐like matrix, was disrupted at high pH or in the presence of organic solvents indicates that it is feasible to use poly(2,7‐DHN) to encapsulate organic compounds and to trigger their release by also changing the external environment. Encapsulation of drugs by polymeric aggregates and their pH‐dependent release have proven to be effective for medical applications (Schmaljohann, 2006). The extent of such release by a pH change within a physiologically relevant range, however, remains to be evaluated in detail for our poly(2,7‐DHN) system. We also compared the coating robustness of poly(2,7‐DHN) with that of polydopamine, the mussel‐inspired polycatechol agent. In contrast to poly(2,7‐DHN), polydopamine was found to be stable in the presence of organic solvents (Fig. 2A and B). To evaluate any effect of particle size distribution of the coating agents on this difference in the robustness between poly(2,7‐DHN) and polydopamine, we measured the hydrodynamic size distribution of the agents. The average diameters were observed to be 30.5 μm for polydopamine and 35.8 μm for poly(2,7‐DHN) (Fig. S5), indicating that particle size was not a main factor for the difference. A detailed evaluation of binding forces of catechol and of 2,7‐DHN by using a surface force apparatus (Hwang *et al*., 2012) would be a key method to interpret the difference in robustness between poly(2,7‐DHN) and polydopamine.

**Figure 3:**
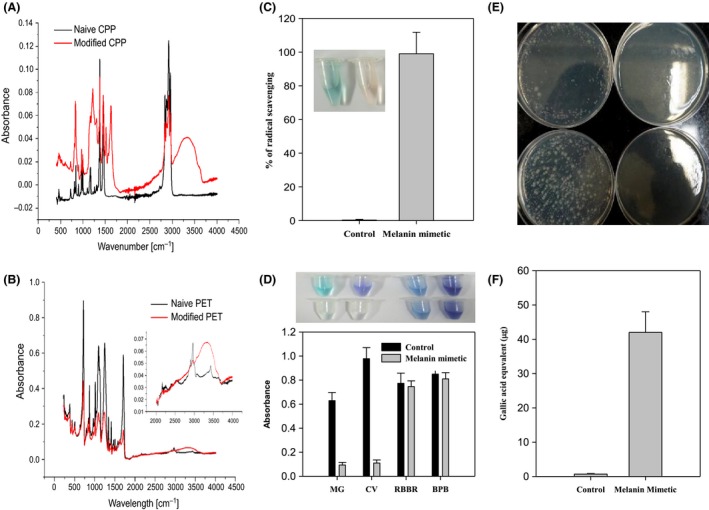
FTIR‐ATR spectra of 2,7‐DHN‐coated A. CPP and B. PET. C. Radical scavenging and D. selective sorption behaviour of 2,7‐DHN‐derived polymer (photograph, left to right: malachite green (+); crystal violet (+); Remazol Brilliant Blue R (−); Bromophenol blue (−), top to down: control; poly(2,7‐DHN) treated). E. Anti‐bacterial (left to right: control; DHN‐derived polymer, top to down: 10^−3^; 10^−2^ dilution) activity and F. phenol content of 2,7‐DHN‐derived polymer.

To confirm that the poly(2,7‐DHN) bore multiple hydroxyphenyl groups, we employed several different experimental methods. Fourier transform infrared‐attenuated total reflectance (FTIR‐ATR) measurements were taken on poly(2,7‐DHN)‐coated PET and CPP surfaces. The coated layer gave peaks at 3200–3400 cm^−1^, corresponding to a high concentration of phenol groups (Fig. 3A and B). Also, since hydroxyphenyl groups have been linked to reactive radical scavenging and bactericidal activity, and such properties have been readily observed in polyphenolics derived from plant biomass (Wang and Ho, 2009; Daglia, 2012), we employed the 2,2′‐azino‐bis(3‐ethylbenzothiazoline‐6‐(sulfonic acid)) (ABTS) radical and *Escherichia coli* to determine whether the poly(2,7‐DHN) exhibits similar properties. The addition of poly(2,7‐DHN) particulates rapidly induced decolouration of the blue‐coloured ABTS radical, indicating that the radical was effectively scavenged (Fig. 3C). This finding is consistent with the *in vivo* physiological roles of fungal melanin in pathogenesis. The pigment present in the cell wall can contribute to reduction of the oxidative burst capacity of the host immune system via its reactive oxygen scavenging action (Jeon and Chang, 2013b). *E. coli* proliferation was also effectively inhibited in the presence of poly(2,7‐DHN) (Fig. 3E). These results both strongly support the hypothesis that poly(2,7‐DHN) consists of a hydroxyphenyl group‐bearing polyaromatic. Further evidence for this hypothesis was obtained from analysis of the charge‐dependent sorption behaviour of the polymer. The material was found to adsorb cationic dyes but not anionic dyes (Fig. 3D). This sorption pattern was attributed to non‐covalent interactions between the polymer and dye, with the hydroxyphenyl groups being transformed into negatively charged moieties or providing negative dipole moments that would interact preferentially with cationic organic molecules. Furthermore, the phenolic groups may exert a repulsive force on anionic species. The determined *Zeta* potential value of −2.51 mV (see Experimental section) of the poly(2,7‐DHN) also supported this preferred sorption of cationic charges. To achieve a more direct indication of the phenol content of poly(2,7‐DHN), a phenol‐reactive reagent (i.e. Folin‐Ciocalteu reagent) was employed, and a significant signal was observed, verifying the presence of phenolic groups (Fig. 3F).

Hydroxyphenyl moieties chelate several metal ions, with subsequent reduction facilitating surface metallization (Lee *et al*., 2007). In addition, the versatile binding properties of polyphenolic coating layers promote the capture of organic compounds, which can be further employed to form secondary layers. These secondary reactions are initiated from the polyphenolic layers, allowing the coated surface to exhibit additional desirable functionalities. In addition to post‐immobilization processes, co‐incubation of functional organic molecules (e.g., cell growth and differentiation factors) during *in situ* polymerization of dopamine has also proven to be effective for surface functionalization. Co‐immobilized organic species can also induce secondary reactions such as silicification and atom transfer radical polymerization (Kang *et al*., 2012).

**Figure 4:**
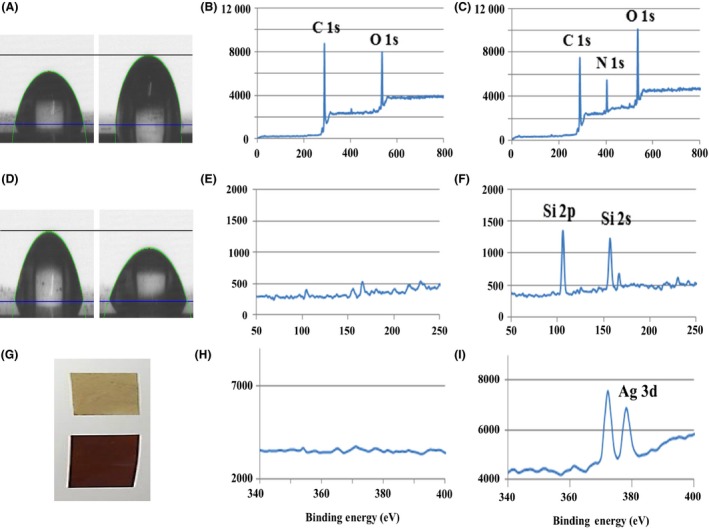
A. Water contact angle of PET film before (left) and after (right) bovine serum albumin (BSA) post‐immobilization. XPS spectra of the film B. before and C. after BSA post‐immobilization. D. Water contact angle of PET film coated with 2,7‐DHN with a precursor, 2‐dimethylaminoethanethiol for co‐immobilization and further silicification. Left to right: before silicification; after silicification. Si peaks revealed by XPS analysis E. before silicification or F. after silicification. G. Photograph of electroless silver metallization of 2,7‐DHN‐coated PET film. Top to down: Before and after metallization. Silver (Ag 3d) XPS peaks of PET film H. before and I. after the metallization.

In the present study, the observation of hydroxyphenyl groups in poly(2,7‐DHN) strongly indicates the feasibility of post‐modification of a layer made of this polymer. To test this suggestion, we used bovine serum albumin (BSA), which is known to enhance the compatibility of blood with various surfaces (Wei *et al*., 2010). By applying a simple dipping method, proteins could be attached to the polyphenolic groups of poly(2‐7‐DHN). This procedure resulted in an effective modification of the surface, which was verified by the observed change in the water contact angle (Fig. 4A, Table S4). X‐ray photoelectron spectroscopy (XPS) provided further evidence for the successful modification, with a clear nitrogen peak due to the conjugated protein present in the spectrum of the modified surface (Fig. 4B and C). In the case of dopamine‐based coatings, a Schiff base between lysine groups of BSA and catechol groups of polydopamine (i.e., chemisorption) is readily formed through quinone formation from the catechol (Wei *et al*., 2010; Lynge *et al*., 2011). However, 2,7‐DHN does not form the corresponding quinone groups theoretically, thus excluding the possibility of chemisorption between BSA and poly(2,7‐DHN). In our conditions, the polyphenolic groups on poly(2,7‐DHN) layers might capture BSA proteins physically. It is noticeable that physical interactions between polyphenols and proteins have been frequently reported (Xiao and Kai, 2012).

The tertiary amine, 2‐(dimethylamino) ethanethiol, which is known to initiate biological silicification (Kim *et al*., 2004; Kang *et al*., 2012), was utilized to confirm whether poly(2,7‐DHN) could co‐immobilize with functional organic molecules. Induction of silicification by the immobilized tertiary amine was verified from the increase in the hydrophilicity of the modified surface (Fig. 4D, Table S4), which is consistent with previous reports on silicification (Kang *et al*., 2012). More direct evidence was obtained from surface analysis using XPS. Significant silicon peaks (i.e., Si 2p and Si 2s) were only detected on the surfaces co‐incubated with the amine compound (Fig. 4E and F).

Finally, we confirmed that the poly(2,7‐DHN) layer was able to promote the metallization of solid surfaces with silver ions. Silver deposition on the polymer coating was identified by the significant change in the colour of the surface (Fig. 4G), as well as by XPS analysis (Fig. 4H and I). The results also indicated that the poly(2,7‐DHN)‐derived polyphenolic moieties were able to directly reduce metal ions, providing the potential for nanoparticle synthesis without the need for additional reducing agents.

Some of the physicochemical properties of poly(2,7‐DHN) reported in Figs 3 and 4 are of great interest for investigators involved in microbial biotechnology. The bactericidal activity of poly(2,7‐DHN) combined with surface engineering can allow for the manufacturing of anti‐bacterial surfaces. In addition, both malachite green and crystal violet are mutagenic agents in water, supporting that poly(2,7‐DHN) can be applicable to a bioremediation strategy by surface modification with some adsorbent agents exhibiting large surface areas. It is also reasonable to expect that the ability of poly(2,7‐DHN) to chelate silver ions can be extended to the chelation of other, toxic heavy metals such as Cr^6+^ and Pb^2+^. Finally, the radical scavenging activity of poly(2,7‐DHN) observed in *in vivo* fungal melanin can be also connected to cellular surface engineering of living fungi, as clearly demonstrated in artificial yeast encapsulation with polydopamine (Yang *et al*., 2011).

## Conclusions

**Scheme 2:**
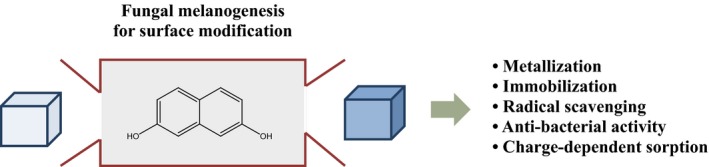
Oxidative polymerization of 2,7‐dihydroxynaphthalene (DHN) leads to material‐independent nanothickness coating. The coated surface is further linked to metallization and immobilization. Innate physicochemical properties of poly(2,7‐DHN) give also rise to surface functionalization showing charge‐dependent sorption, radical scavenging and anti‐bacterial activity.

Here, we describe the application of a simple dip‐coating method for a diverse range of solid substrates by using 2,7‐DHN to mimic fungal melanogenesis. Reacting laccase with 2,7‐DHN led to efficient oxidative polymerization and gave rise to polyaromatics containing multiple hydroxyphenyl groups. The co‐incubation of functional organic molecules during the DHN polymerization resulted in their co‐immobilization onto the surface of the substrates. In addition, hydroxyphenyl groups in the poly(2,7‐DHN) layer could act as adhesive sites for proteins or chelating/reductive sites for electroless metallization, thus contributing to secondary surface functionalization (Scheme 2).

The study demonstrates that a unique mussel‐inspired, material‐independent adhesion is possible with polyphenolic moieties, even in the absence of a polycatechol structure. Furthermore, structural features (i.e. polyaromaticity and hydroxyphenyl group), which lead to MAP‐like adhesion, is identifiable in other biological species. This suggests that a similar material platform is used for several *in vivo* functions, including adhesion, radical scavenging and pigmentation, which are critically linked to the viability of biological species.

## Experimental procedures

### Materials

Stainless steel (Nalclip^®^, Buyhearts, Seoul, Korea), aluminium (15 μm; Daihan Eunpakgy, Asan, Korea), PET (188 μm; Mitsubishi, Tokyo, Japan), CPP (40 μm; Sammin Chem, Seoul, Korea), nylon (15 μm; Daihan Eunpakgy) and glass (microscope cover slides, Marienfeld, Lauda‐Königshofen, Germany) were obtained for surface modification. Cellulose acetate was obtained from a cigarette filter (ONE^®^, KT&G, Daejon, Korea). Plant leaf (*Heteropanax fragrans*) and fine granite were obtained from a local flower shop located in Daejeon, Korea. ABTS and bromophenol blue sodium salt were purchased from Fluka and USB, respectively. Other chemicals (i.e. *Trametes versicolor* laccase, sodium acetate (anhydrous), glacial acetic acid, tetramethyl orthosilicate (TMOS), BSA, 2‐(dimethylamino) ethanethiol hydrochloride, dopamine hydrochloride, Folin & Ciocalteu's phenol reagent, malachite green oxalate salt, crystal violet, Remazol Brilliant Blue R, sodium carbonate, silver nitrate, 2,7‐DHN, dopamine hydrochloride and gallic acid) were obtained from Sigma‐Aldrich (St. Louis, MO, USA). Laccase activity was measured as described previously (Jeon *et al*., 2010). Commercial *T. versicolor* laccase exhibited an activity of 9.34 × 10^6^ U mg^−1^.

### Dip‐coating for one‐pot surface modification

2,7‐DHN (5 mg/ml) was completely dissolved in 32 ml of 100 mM sodium acetate buffer (pH 5.0) and 8 ml of absolute methanol in a 50 ml conical tube, and then the solution was poured into a square petri dish containing the solid substrates. The dish was subsequently incubated at room temperature with gentle shaking. For polymerization of the DHN, *T. versicolor* laccase (0.1 mg/ml) was added to the coating solution prior to incubation. The coated surfaces were gently rinsed with distilled water and dried at room temperature. Dopamine coating was performed as described previously (Jeon *et al*., 2013a).

### Coating robustness test

The colour values (L*, a* and b*) of coated PET film sheets were measured using a chromameter (COH‐400; Nippon Denshoku Tokyo, Japan). They were then completely immersed in 1 N sodium hydroxide, 1 N hydrochloric acid and 1 N sodium chloride solution or 100% methanol, acetone, 2‐propanol and 1,4‐dioxane for 4 h. The samples were rinsed with distilled water and dried at room temperature. Colour values of the completely dried PET films were again measured. The colour change (ΔE) on soaking was calculated using the equation: ΔE = [(100 − L*)^2^ + (a*)^2^ + (b*)^2^]^1/2^.

### Hydrodynamic size distribution and Zeta potential measurements of poly(2,7‐DHN)

2,7‐DHN‐derived melanin solutions were obtained through the dip‐coating conditions for 34 h. Hydrodynamic size distribution and Zeta potential value were evaluated using an electrophoretic light scattering spectrophotometer (ELS 8000; Otsuka, Osaka, Japan). Polydopamine was synthesized for 34 h as described previously (Jeon *et al*., 2013a).

### Physicochemical properties of poly(2,7‐DHN)

2,7‐DHN (5 mg/ml) was dissolved in 20% methanol in distilled water, and then laccase (0.1 mg/ml) was added in order to induce polymerization. After 36‐h incubation, the suspended polymeric product could be readily observed with the naked eye. Repeated centrifugation (13 000 r.p.m.) and washing with distilled water were performed for collection of the product.

Charged dyes were used to evaluate the specific sorption behaviour of the polymeric product. Predefined amounts of individual dyes were dissolved in distilled water to give solutions with absorbances ranging from 0.5 to 1.0 at the λ_max_ of each dye (i.e. 620 nm for malachite green, 580 nm for crystal violet, 595 nm for Remazol Brilliant Blue R and 590 nm for bromophenol blue). The dye‐containing water was mixed with the polymeric product suspended in distilled water (1 mg of dry weight) with vigorous vortexing for 30 s and then centrifuged to collect the treated polymeric product. The absorbances of the supernatants were measured at the λ_max_ of each dye.

ABTS (10 mM) in distilled water (10 ml) was reacted with laccase enzyme (50 μg/ml) to give stable ABTS radicals. After significant colour change of the reaction media from colourless to dark blue, ultracentrifugation using a membrane filter (5000 NMWL filter unit, Millipore Ultrafree^®^‐MC Merck Millipore, Darmstadt, Germany) was performed to exclude the treated enzyme. The pure ABTS radical was then diluted with distilled water to give an absorbance of 1.0 at 420 nm. The polymeric product suspended in distilled water (1 mg of dry weight) was poured into the ABTS radical‐containing water (950 μl) and vigorous vortexing was then performed for 30 s. Finally, centrifugation (13 000 r.p.m.) was performed to exclude the treated polymeric product, and the absorbance at 420 nm of the supernatant was measured using a UV‐visible spectrophotometer (Cary 3‐Bio; Varian, Palo Alto, CA, USA). The percentage radical scavenging was calculated using the following equation:

$$\%\text{radical scavenging}=100-(100\times S_{A})$$

where *S*_*A*_ is the absorbance of the samples.

To evaluate the total phenolic content, the polymeric product suspended in distilled water (2.5 mg of dry weight) was mixed with 2.5 ml of 0.2 N Folin‐Ciocalteu reagent for 5 min, followed by the addition of 2 ml of 75 g/l sodium carbonate. After 2‐h incubation at room temperature, the absorbance at 760 nm was measured using a UV‐visible spectrophotometer. Gallic acid was used to produce a calibration curve.

*Escherichia coli* XL1 Blue was used to assess whether the polymeric product exhibits anti‐bacterial activity. The bacteria were grown in LB broth (100 ml) for 12 h, and 2 ml of the suspension was then centrifuged at 13 000 r.p.m. The collected *E. coli* was washed three times with phosphate‐buffered saline (PBS, pH 7.0) and then serially diluted in the range of 10^−1^ to 10^−6^. All diluted samples were mixed with the polymeric product suspended in distilled water (1 mg of dry weight) and incubated with gentle shaking for 72 h. Following this, 50 μl of the samples was spread on LB‐containing agar plates and incubated at 38°C for 24 h. Colony‐forming units of the samples were then compared with those of the corresponding controls.

### Post‐ and co‐immobilization experiments

2,7‐DHN‐coated PET films were immersed in a solution of 5 mg/ml BSA in PBS (pH 7.8). After 15 h, the BSA‐immobilized film was washed with distilled water and dried at room temperature. For co‐immobilization experiments, the PET film was initially immersed in the DHN solution (40 ml) for 1 h at room temperature. After this, 20 ml of the liquid was removed and replaced with 16 ml of 100 mM sodium acetate buffer (pH 5.0) containing 2‐(dimethylamino) ethanethiol hydrochloride (36 mM) and 4 ml of absolute methanol. Further incubation was performed for 7 h, and the film was then rinsed with distilled water and dried at room temperature. Monosilicic acid was formed by stirring a 1 mM HCl solution of TMOS (100 mM) for 15 min at room temperature. A 20‐ml sample of this was then mixed with 100 mM aqueous phosphate buffer (pH 6.0, 20 ml). The 2‐(dimethylamino) ethanethiol‐coated PET film was subsequently incubated in this solution for 1.5 h and then washed with distilled water and dried at room temperature.

### Surface characterization of 2,7‐DHN‐coated films

For SEM analysis of 2,7‐DHN coated layers (JSM‐7610F; JEOL, Tokyo, Japan), we coated polystyrene‐based plastic surfaces with the one‐pot coating conditions for 24 h. An XPS (ESCALAB 250, Thermo VG Scientific, West Sussex, UK) instrument using Al K_α_ (1486.6 eV) as a radiation source was employed to determine the composition of the coated PET films. The take‐off angle of the photoelectrons was set as 90° for the measurements. FTIR‐ATR spectra of the coated films were obtained using a 660‐IR spectrometer (Varian). The hydrophilicity of the coated films was characterized indirectly by static water contact angle measurement using a contact angle goniometer (DSA 100; KRUSS GmbH, Hamburg, Germany) equipped with video camera. A 3‐μl droplet of distilled water was placed on the film surfaces at room temperature, and the angle was measured after 3 s. Six measurements were averaged for each surface to achieve a reliable value. The thicknesses of the coatings on PET were measured using atomic force microscopy (AFM; Veeco, Santa Barbara, CA, USA). Half of the coated area was placed in contact with 1 N sodium hydroxide solution for 10 s to detach the polymeric coating followed by AFM scanning to measure the height difference between the detached and the coated layers. Three different coated regions were measured to obtain average and standard deviations.

### Electroless metallization

Silver deposition was carried out as previously described (Lee *et al*., 2007). The coated PET surfaces were completely immersed in a 100 mM aqueous silver nitrate solution for 72 h at room temperature. They were then washed with distilled water and dried at room temperature.

### Mass spectrometric analysis of laccase‐mediated polymerization of 2,7‐DHN

Electrospray ionization‐mass spectrometry coupled with CID (Collision‐induced dissociation) MS/MS (API 2000; Applied Biosystems Foster City, CA, USA) was performed in negative mode (−4000 V) to confirm whether the 2,7‐DHN underwent homo‐polymerization to form the material‐independent coating. Deionized water was used to prepare the reaction solutions in order to prevent salts from causing inaccuracies in the m/z measurements. After 24 h of reaction, 0.5 ml of each sample was filtered through a 0.45 μm syringe filter (Millipore PTFE type) and then mixed with 0.5 ml of pure acetonitrile. Before analysing the reaction samples, we first obtained CID MS/MS data for the monomer. We then compared the CID MS/MS fragmentation patterns of homo‐oligomer ions with those of the standard monomer to elucidate the monomer composition of the oligomer ions.

### Prep‐LC and NMR analyses

Reaction products after 2 h were extracted with ethyl acetate and then separated with reverse‐phase HPLC. HPLC was performed in Agilnet 1260 infinity LC equipped with a diode array detector and a ZORBAX SB C‐18 column at 25°C, with an aqueous solvent system (flow rate, 0.6 ml/min) containing 35% acetonitrile. Absorbance was monitored at 254 nm. The separated fractions were dissolved in acetone‐d^6^. NMR was performed with Bruker Avance III HD 700 MHz NMR spectrometer.

^1^H NMR (acetone‐d^6^) for Fraction 3: δ 6.23 (d, J = 2.3 Hz, H5), δ 6.78 (dd, J = 8.8, 2.5 Hz, H4), δ 6.97 (d, J = 8.8, 2.5 Hz, H1), δ 7.61 (d, J = 8.8, H3), δ 7.65 (d, J = 8.8, H2).

^1^H NMR (acetone‐d^6^) for Fraction 4: δ 6.48 (d, J = 2.4 Hz, H10), δ 6.77, δ 6.85 (m, H1, H9, H11), δ 6.93 (d, J = 8.8 Hz, H4), δ 6.97 (d, J = 2.3 Hz, H5), δ 7.06 (s, H6), δ 7.43 (s, H7), δ 7.57 (m, H2, H3, H8).

## Conflict of interest

None declared.

## Supporting information


**Fig. S1.** MS and MS/MS spectra of *in vitro* laccase‐catalysed polymerization of 2,7‐DHN. (A) MS spectrum of the enzymatic reaction; (B) Collision‐induced dissociation (CID) MS/MS of 317 m/z; (C) CID MS/MS of 475 m/z.
**Fig. S2.** H^1^ NMR spectroscopic analysis of two major intermediates during laccase‐catalysed 2,7‐DHN polymerization.
**Fig. S3.** SEM image of 2,7‐DHN‐coated polystyrene plastic surfaces: (A) untreated and (B) treated.
**Fig. S4.** AFM image of 2,7‐DHN‐coated PET film for coating thickness measurement. Half region of the coated PET was soaked in 1N NaOH for 10 s, thus making the DHN layer totally detached. The height difference between the detached and the coated PET surfaces was monitored through AFM scanning.
**Fig. S5.** Hydrodynamic size distribution of product of the laccase‐catalysed polymerization of 2,7‐DHN.
**Table S1.** Comparison of relevant collision‐induced dissociation (CID) MS/MS peaks of homo‐oligomers formed in *in vitro* laccase‐mediated polymerization of 2,7‐DHN with those of standard 2,7‐DHN.
**Table S2.** Time course of changes in coating thickness of 2,7‐DHN coated PET film.
**Table S3.** Water contact angle of 2,7‐DHN‐coated films. Average and standard deviations of six measurements were shown.
**Table S4.** Water contact angle of post‐ or co‐immobilized PET films shown in Fig. 4. Average and standard deviations of six measurements were shown.
