# Supplementary material for: Dihydroxynaphthalene‐based mimicry of fungal melanogenesis for multifunctional coatings
Source: Microb Biotechnol. 2016 Feb 2;9(3):305–15. doi: 10.1111/1751-7915.12347 (PMC4835569; doi:10.1111/1751-7915.12347)
Supplement: Supplementary file 1 — Fig. S1. MS and MS/MS spectra of in vitro laccase‐catalysed polymerization of 2,7‐DHN. (A) MS spectrum of the enzymatic reaction; (B) Collision‐induced dissociation (CID) MS/MS of 317 m/z; (C) CID MS/MS of 475 m/z. Fig. S2. H1 NMR spectroscopic analysis of two major intermediates during laccase‐catalysed 2,7‐DHN polymerization. Fig. S3. SEM image of 2,7‐DHN‐coated polystyrene plastic surfaces: (A) untreated and (B) treated. Fig. S4. AFM image of 2,7‐DHN‐coated PET film for coating thickness measurement. Half region of the coated PET was soaked in 1N NaOH for 10 s, thus making the DHN layer totally detached. The height difference between the detached and the coated PET surfaces was monitored through AFM scanning. Fig. S5. Hydrodynamic size distribution of product of the laccase‐catalysed polymerization of 2,7‐DHN. Table S1. Comparison of relevant collision‐induced dissociation (CID) MS/MS peaks of homo‐oligomers formed in in vitro laccase‐mediated polymerization of 2,7‐DHN with those of standard 2,7‐DHN. Table S2. Time course of changes in coating thickness of 2,7‐DHN coated PET film. Table S3. Water contact angle of 2,7‐DHN‐coated films. Average and standard deviations of six measurements were shown. Table S4. Water contact angle of post‐ or co‐immobilized PET films shown in Fig. 4. Average and standard deviations of six measurements were shown. [file MBT2-9-305-s001.pptx]

## Slide 1
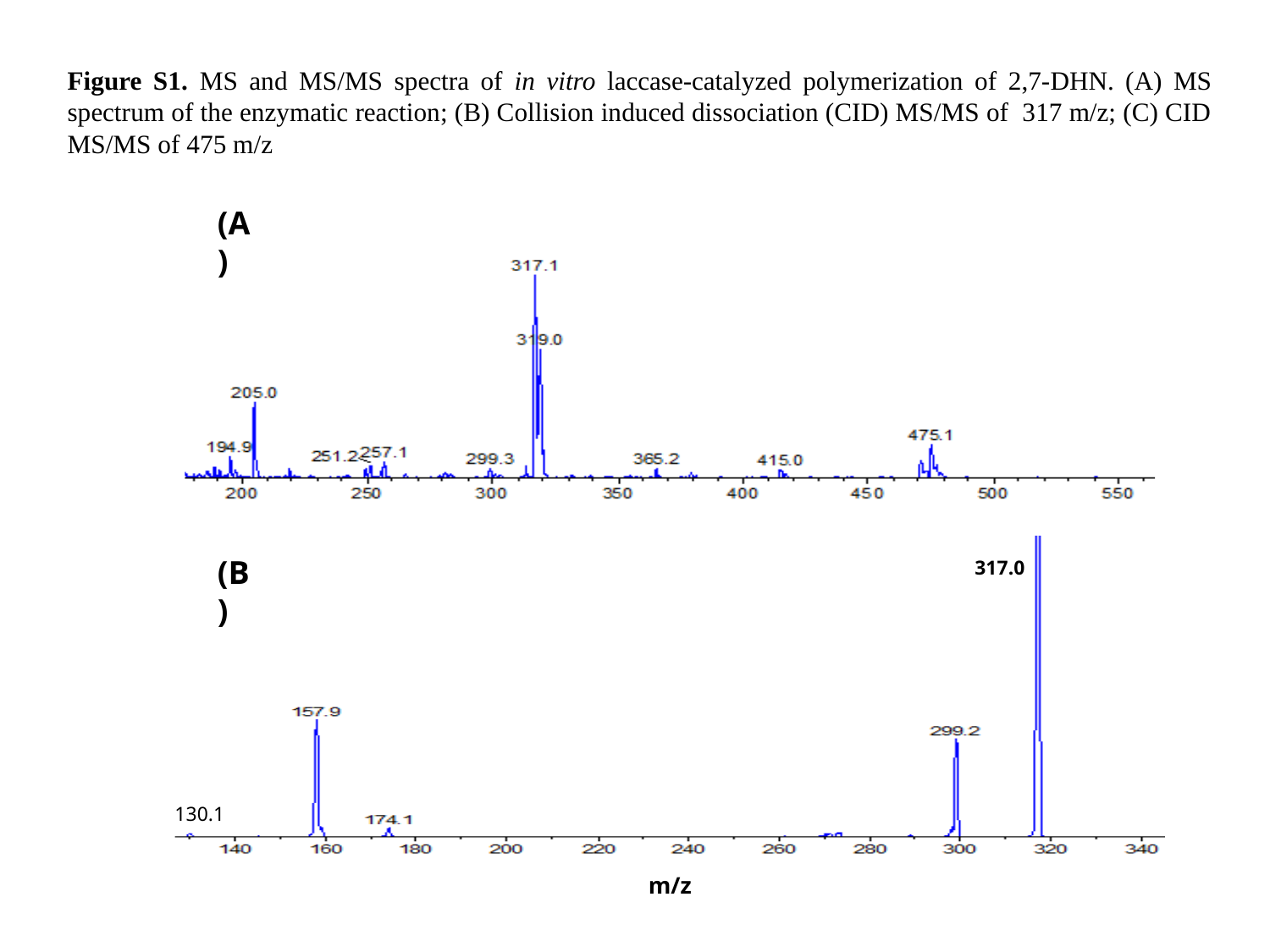

Figure S1. MS and MS/MS spectra of in vitro laccase-catalyzed polymerization of 2,7-DHN. (A) MS spectrum of the enzymatic reaction; (B) Collision induced dissociation (CID) MS/MS of 317 m/z; (C) CID MS/MS of 475 m/z
(A)
(B)
317.0
130.1
m/z

## Slide 2
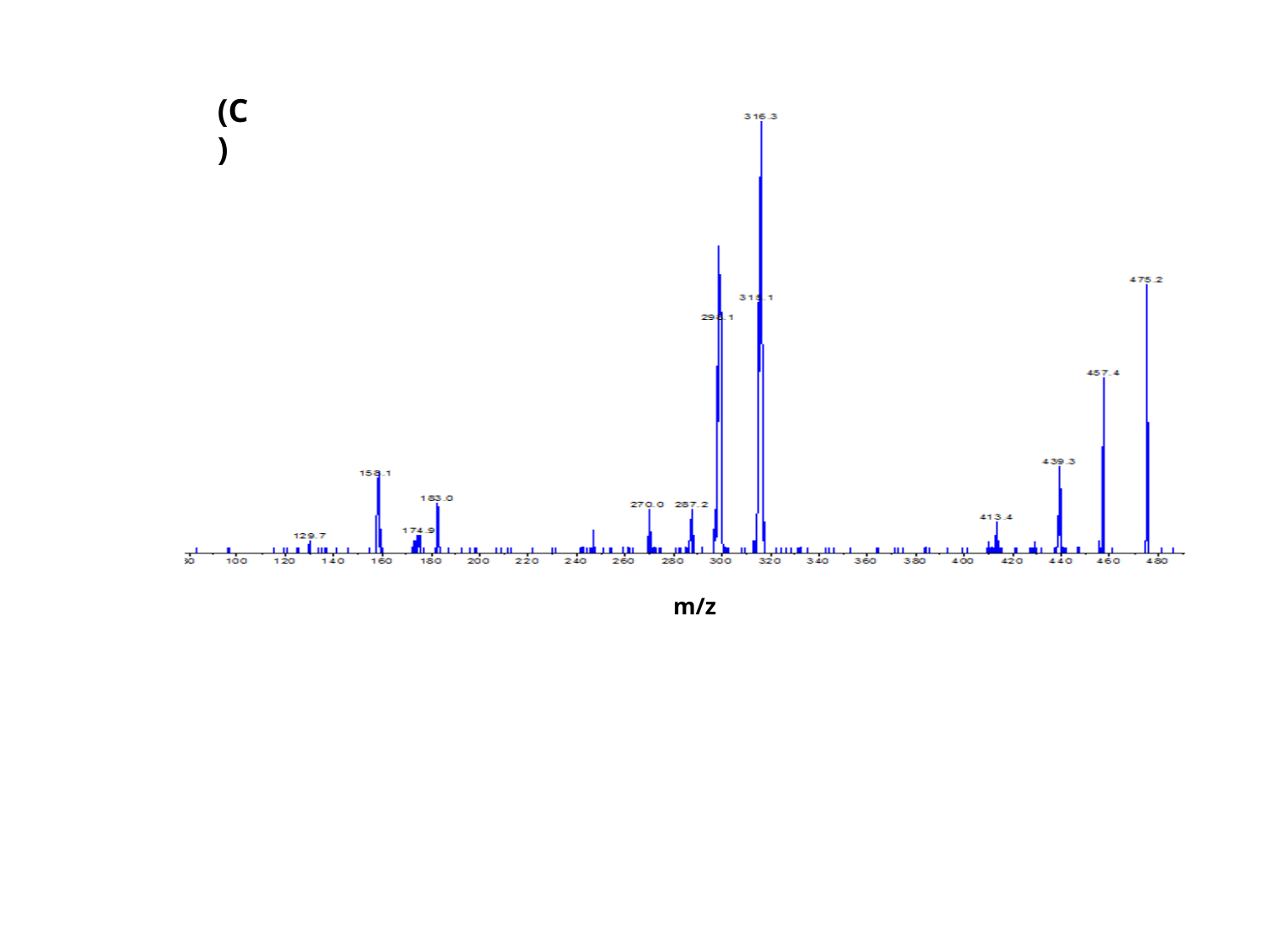

(C)
m/z

## Slide 3
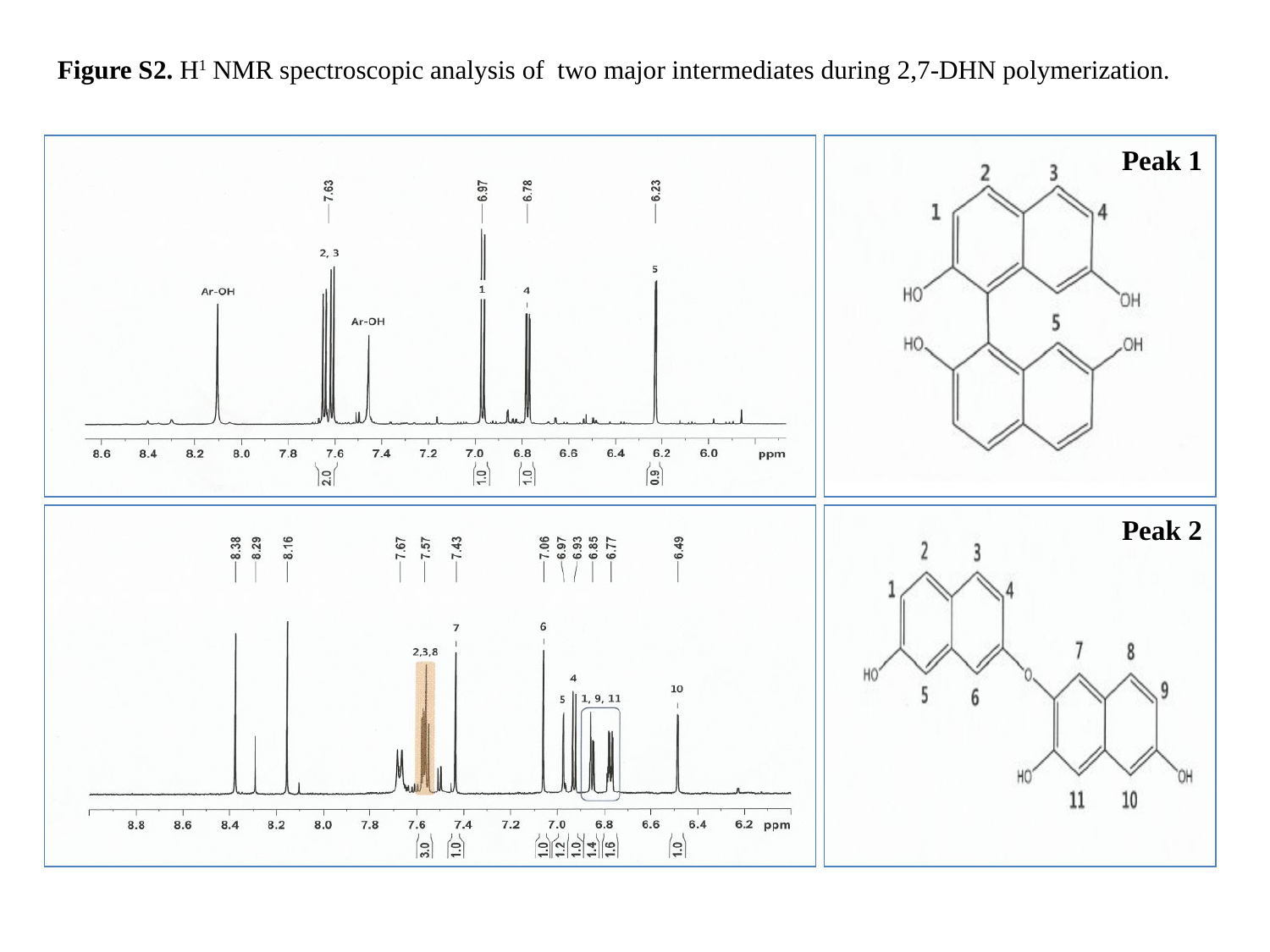

Figure S2. H1 NMR spectroscopic analysis of two major intermediates during 2,7-DHN polymerization.
Peak 1
Peak 2

## Slide 4
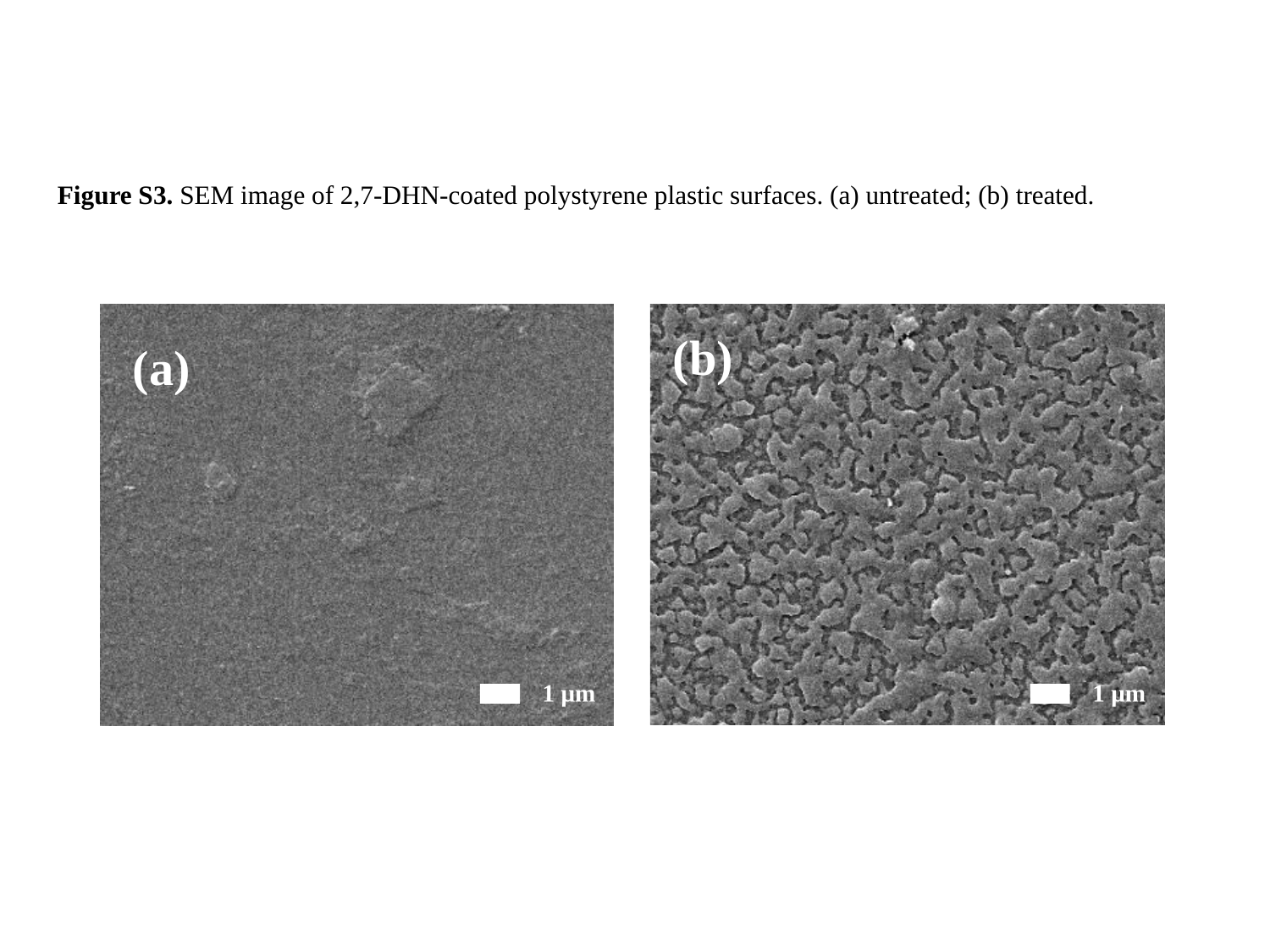

Figure S3. SEM image of 2,7-DHN-coated polystyrene plastic surfaces. (a) untreated; (b) treated.
(b)
(a)
1 μm
1 μm

## Slide 5
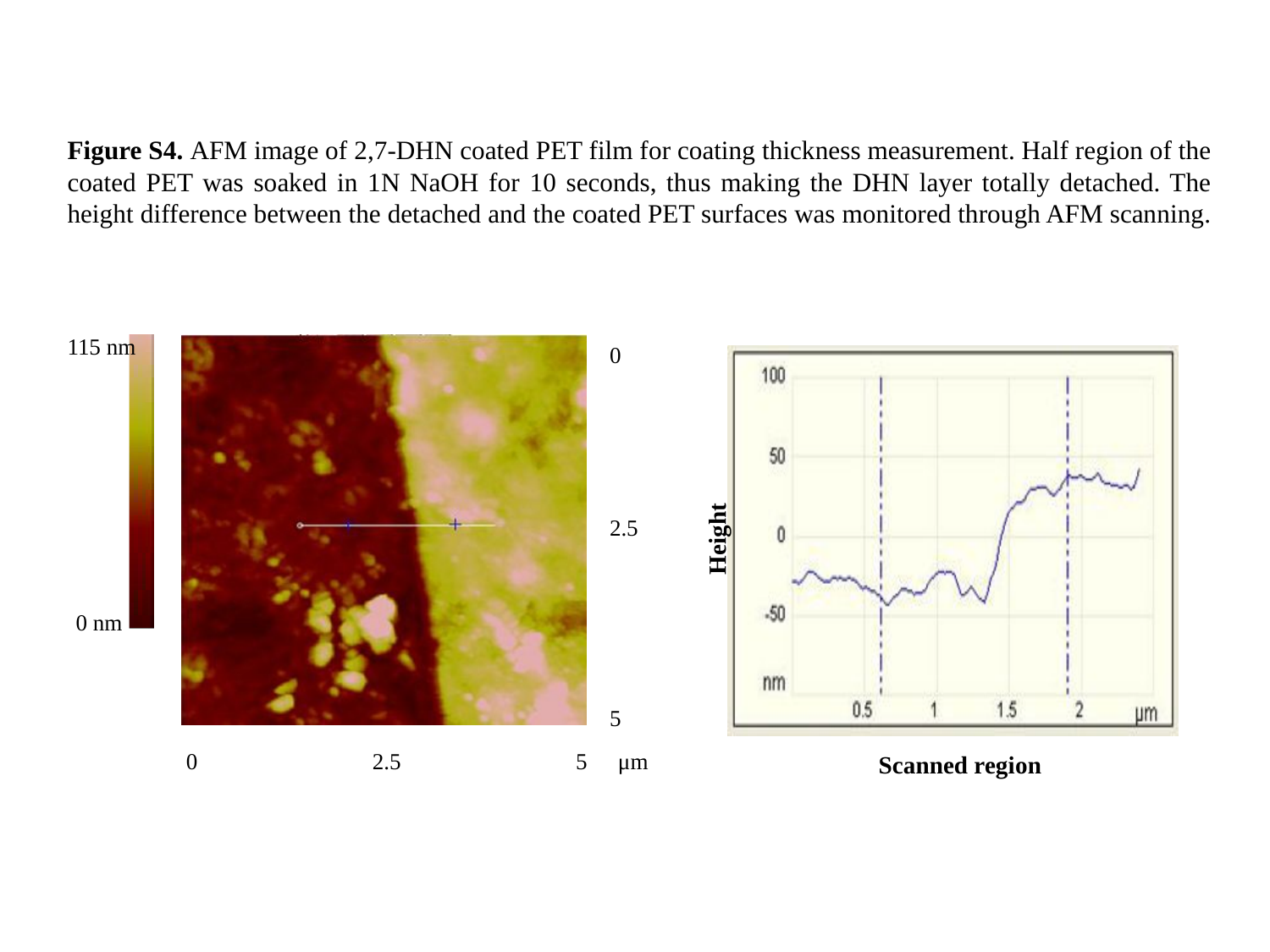

Figure S4. AFM image of 2,7-DHN coated PET film for coating thickness measurement. Half region of the coated PET was soaked in 1N NaOH for 10 seconds, thus making the DHN layer totally detached. The height difference between the detached and the coated PET surfaces was monitored through AFM scanning.
115 nm
0
2.5
Height
0 nm
5
0
2.5
5
μm
Scanned region

## Slide 6
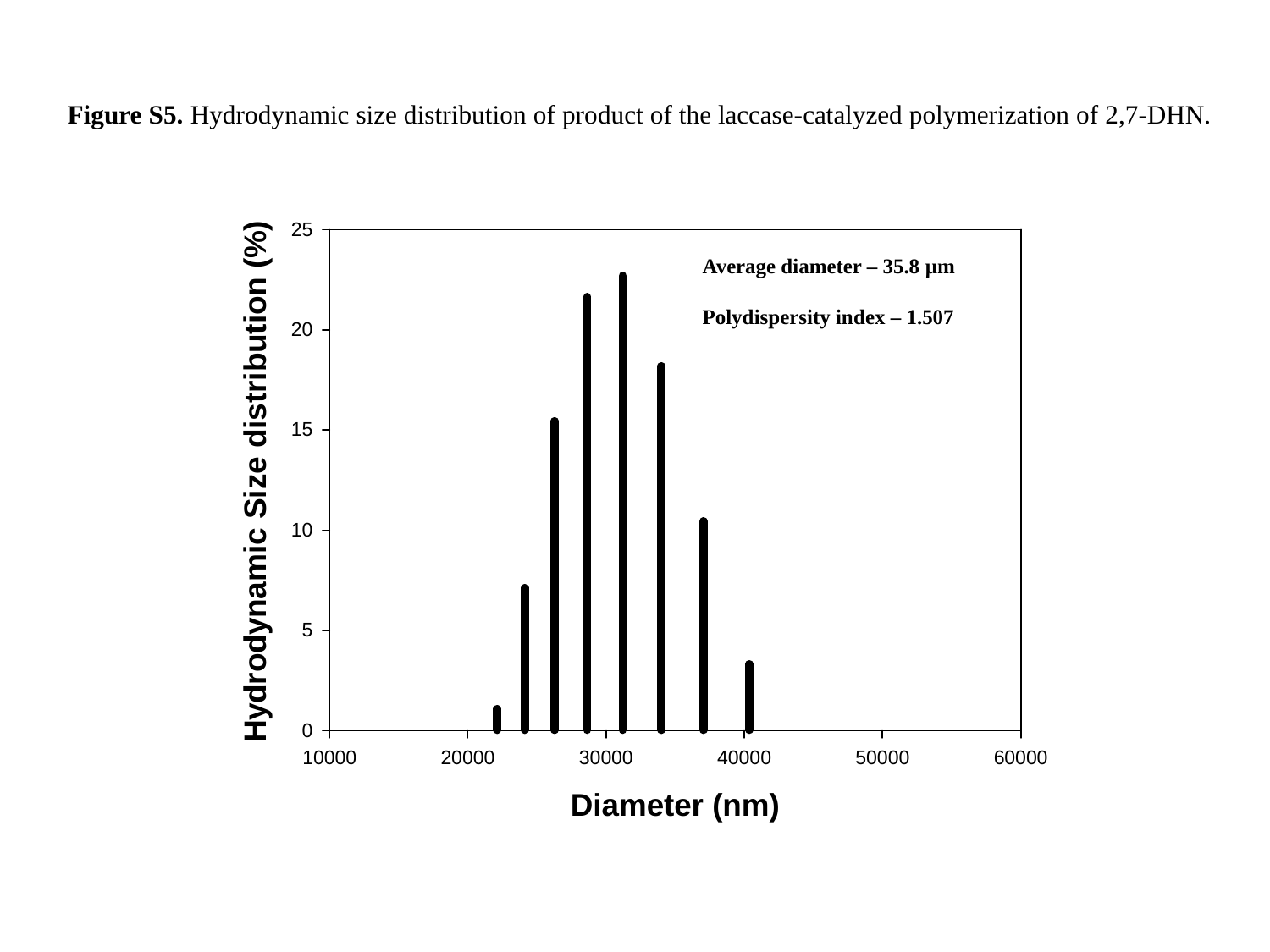

Figure S5. Hydrodynamic size distribution of product of the laccase-catalyzed polymerization of 2,7-DHN.
Average diameter – 35.8 μm
Polydispersity index – 1.507

## Slide 7
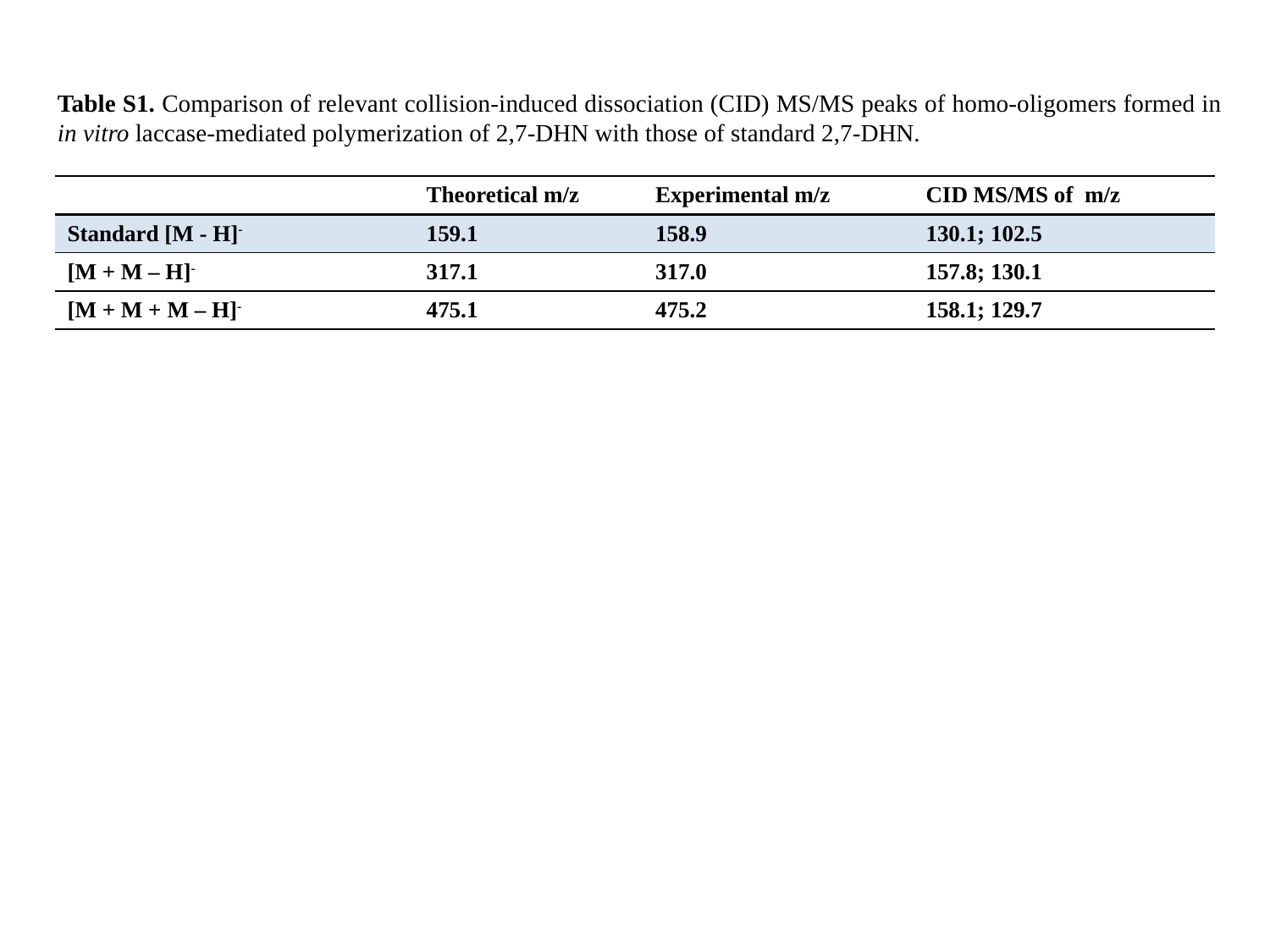

Table S1. Comparison of relevant collision-induced dissociation (CID) MS/MS peaks of homo-oligomers formed in in vitro laccase-mediated polymerization of 2,7-DHN with those of standard 2,7-DHN.
| | | Theoretical m/z | Experimental m/z | CID MS/MS of m/z |
| --- | --- | --- | --- | --- |
| Standard [M - H]- | | 159.1 | 158.9 | 130.1; 102.5 |
| [M + M – H]- | | 317.1 | 317.0 | 157.8; 130.1 |
| [M + M + M – H]- | | 475.1 | 475.2 | 158.1; 129.7 |

## Slide 8
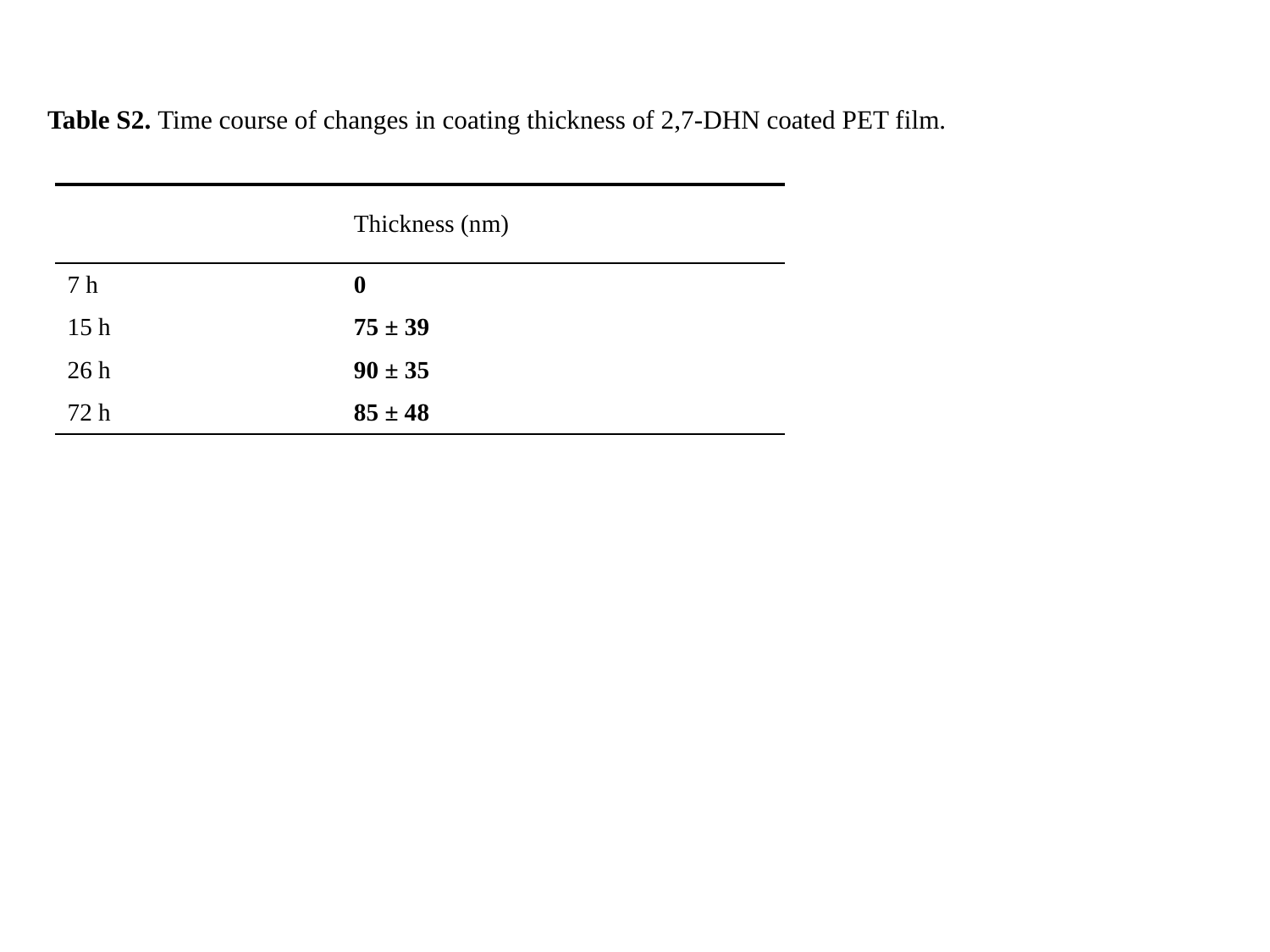

Table S2. Time course of changes in coating thickness of 2,7-DHN coated PET film.
| | Thickness (nm) |
| --- | --- |
| 7 h | 0 |
| 15 h | 75 ± 39 |
| 26 h | 90 ± 35 |
| 72 h | 85 ± 48 |

## Slide 9
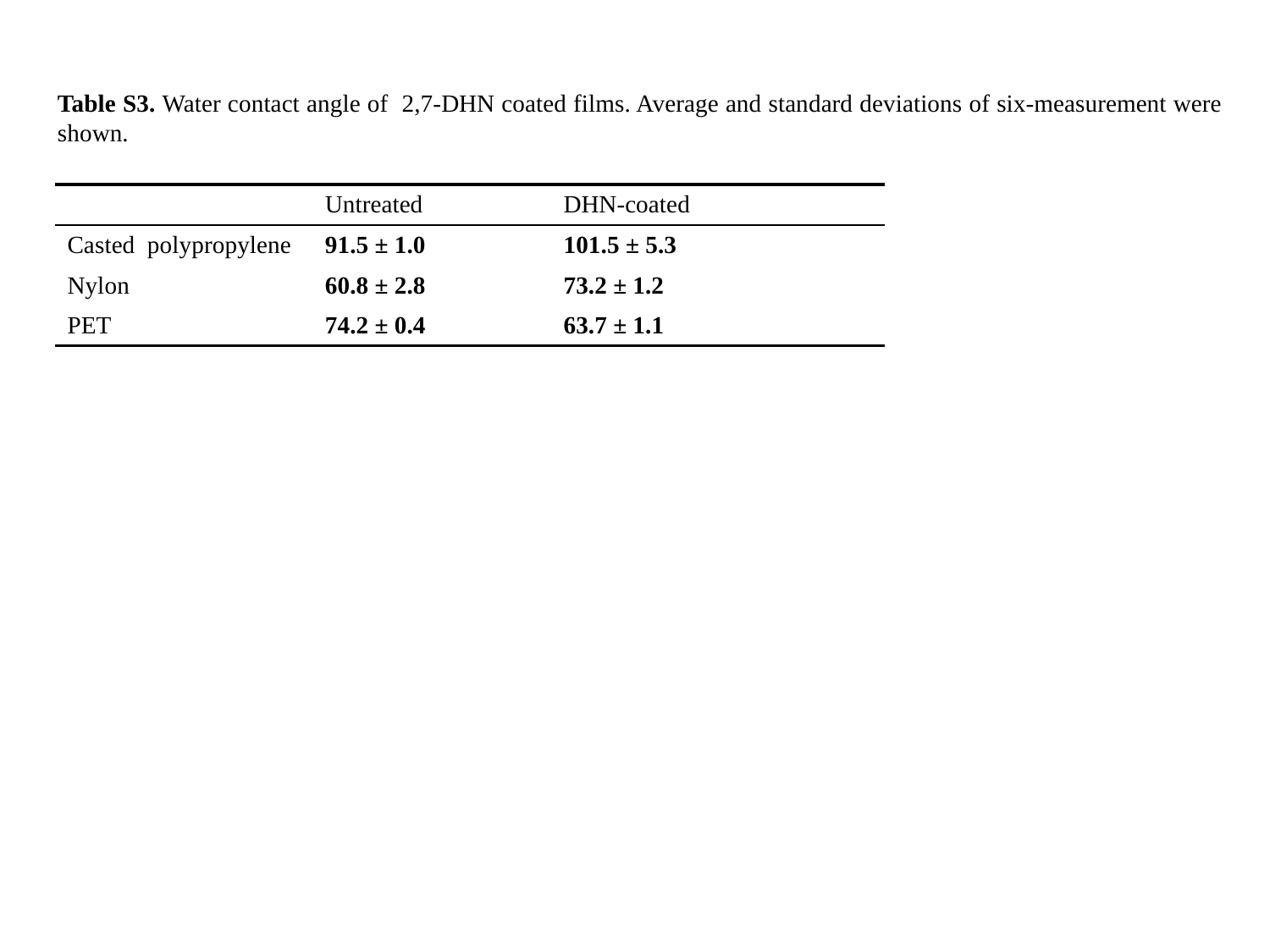

Table S3. Water contact angle of 2,7-DHN coated films. Average and standard deviations of six-measurement were shown.
| | Untreated | DHN-coated |
| --- | --- | --- |
| Casted polypropylene | 91.5 ± 1.0 | 101.5 ± 5.3 |
| Nylon | 60.8 ± 2.8 | 73.2 ± 1.2 |
| PET | 74.2 ± 0.4 | 63.7 ± 1.1 |

## Slide 10
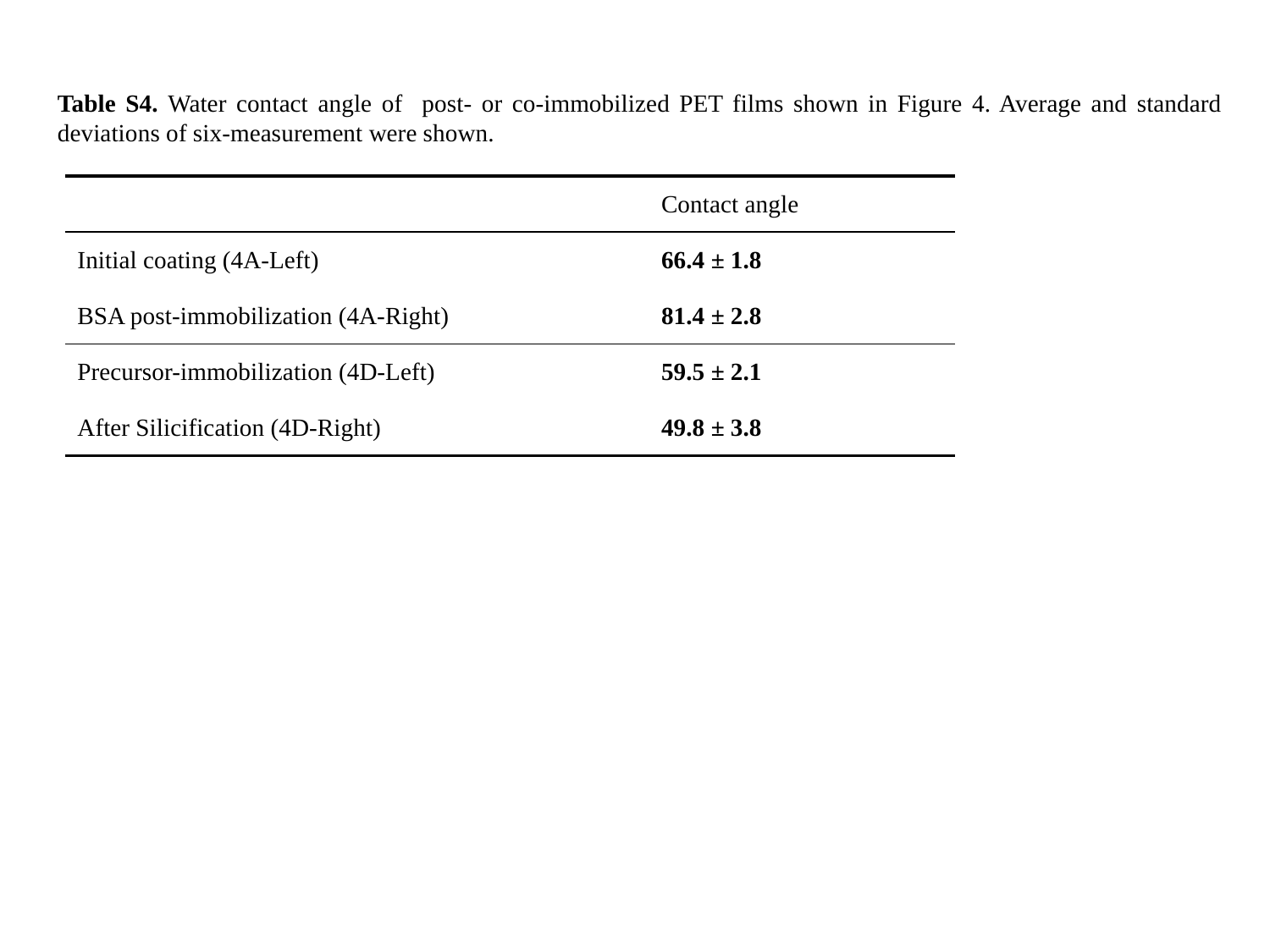

Table S4. Water contact angle of post- or co-immobilized PET films shown in Figure 4. Average and standard deviations of six-measurement were shown.
| | Contact angle |
| --- | --- |
| Initial coating (4A-Left) | 66.4 ± 1.8 |
| BSA post-immobilization (4A-Right) | 81.4 ± 2.8 |
| Precursor-immobilization (4D-Left) | 59.5 ± 2.1 |
| After Silicification (4D-Right) | 49.8 ± 3.8 |
